# Does Fungal Chitosan Leave Noticeable Traces in Treated Wines?

**DOI:** 10.3390/foods13213367

**Published:** 2024-10-23

**Authors:** Margot Paulin, Cécile Miot-Sertier, Julie Miranda, Amélie Vallet-Courbin, Julie Maupeu, Cédric Delattre, Joana Coulon, Virginie Moine, Axel Marchal, Stéphanie Roi, Thierry Doco, Marguerite Dols-Lafargue

**Affiliations:** 1 ISVV and Institute Pascal, University of Bordeaux, INRAE, Bordeaux INP, Bordeaux Sciences Agro, OENO, UMR 1366, ISVV, F-33140 Villenave d’Ornon, France; margot.paulin@hotmail.fr (M.P.); cecile.miot-sertier@u-bordeaux.fr (C.M.-S.); julie.miranda@u-bordeaux.fr (J.M.); axel.marchal@u-bordeaux.fr (A.M.); 2 Microflora-ADERA, UMR 1366, ISVV, F-33140 Villenave d’Ornon, France; amelie.vallet-courbin@u-bordeaux.fr (A.V.-C.); julie.maupeu@u-bordeaux.fr (J.M.); 3 Clermont Auvergne INP, CNRS, Institute Pascal, Université Clermont Auvergne, F-63000 Clermont-Ferrand, France; cedric.delattre@uca.fr; 4 Biolaffort, 11 Rue Aristide Bergès, F-33270 Floirac, France; joana.coulon@laffort.com (J.C.); virginie.moine@laffort.com (V.M.); 5 UMR 1083, UMR Sciences pour l’Oenologie, INRA, SupAgro, UM1, 2 Place Viala, F-34060 Cedex Montpellier, France; stephanie.roi@inrae.fr (S.R.); thierry.doco@inrae.fr (T.D.)

**Keywords:** fungal chitosan treatment, wine, antiseptic, sensorial impact persistence

## Abstract

**Background (1):** The use of fungal chitosan as an antiseptic in wine appears as a promising alternative to sulfur dioxide for the elimination of *Brettanomyces bruxellensis* sensitive strains. Nevertheless, its utilization raises the question, “how are the treated wines different from the untreated ones?” **Methods (2):** Chitosan treatment residues were sought in the oligosaccharide and polysaccharide fractions and among 224 low MW ions (<1800 g·mol^−1^) in several wines by using liquid chromatography (size exclusion HPLC or LC-MS) and GC-MS. Standard oenological parameters were also examined as well as possible sensory modifications by a panel of tasters composed of experts and non-experts. **Results (3):** None of these methods enabled the reproducible and reliable identification of a treated wine without comparing it to its untreated control. The fingerprints of treatment are not reliably detectable by the analytical methods used in this study. However, the treated wines seem permanently protected against the development of chitosan-sensitive strains of *B. bruxellensis*. **Conclusions (4):** If chitosan treatment modifies the wine, the associated changes were not identified by the liquid chromatography method mentioned above and they were not perceived by most people in our taster panel. However, the expected antimicrobial action of chitosan was observed on *B. bruxellensis* sensitive strains and persisted at least one year. Tolerant strains were less affected by these persistent effects.

## 1. Introduction

Wine elaboration necessitates several fermentation stages driven by microorganisms that sequentially contribute to the final quality of the product [1]. However, microorganisms can also alter wine if they develop at the wrong time or if they produce undesirable compounds that affect the wine sensorial properties [2,3]. Often, to avoid these microbial deviations, the winemaker uses antiseptic agents. The most frequently used is sulfur dioxide, both for its efficiency and for the persistence of its activity [4]. Indeed, residual effect are particularly useful and expected during wine aging, as this winemaking step can last several years. Nevertheless, SO_2_ is classified among the priority allergens by the European Union [5] and sometimes, it is not efficient because tolerant strains exist, even in the most feared species [6]. As a result, alternative solutions are becoming more and more considered, among which is chitosan. The use of fungal chitosan as an antiseptic in wine has been authorized since 2009 and 2018 in conventional and organic agriculture, respectively [7,8]. Chitosan is biocompatible (with an ADI equivalent to sucrose, 17 g/Kg of body weight), and its presence in the human body leads to very limited inflammatory reactions and rejection or allergy [9,10,11]. Chitosan inhibits the growth of many microbial species [12]. Microbial inhibition and death result from numerous molecular mechanisms induced by the initial aggregation between the negatively charged microbes and the cationic solubilized chitosan [12,13,14,15,16,17,18,19]. To treat wine contaminated with microbial spoilers, chitosan is directly added to the wine, mixed and, after a period of 3 to 10 days, the wine is racked, eliminating processing residues in the sediment. The main microbial problem solved by chitosan addition is the elimination of *B. bruxellensis* strains tolerant to other treatments such as sulfiting [20,21]. Other wine microbial contaminations may be solved by chitosan treatment. However, the treatment is not always effective [22,23,24,25,26]. In case of treatment of the wine early in the winemaking process, fermentations could be affected. Actually, Castro-Marín et al. [10,27], Scansani et al. [28] and Miot Sertier et al. [22] observed delayed alcoholic or malolactic fermentations in chitosan-treated red wines.

The treatment could therefore leave residues in treated wine. Different hypotheses have been proposed, as follows: (i) some chitosan residues would remain despite racking, or (ii) chitosan treatment may reduce the quantity of nutrients or microelements essential for microbial development of certain species or strains [13]. This leads to the following questions:

- Can fungal chitosan treatment residues be detected or measured through wine chemical composition analyses?
- Is the wine taste significantly modified?
- Is fungal chitosan treatment durable over time? Can chitosan be used preventively in certain situations? In other words, does it have residual effects that would protect the wine in case of new contamination?

In this study, the aim is to answer these questions with a specific focus on microbial stabilization in case of contamination by *B. bruxellensis*.

## 2. Materials and Methods

**Figure 1:**
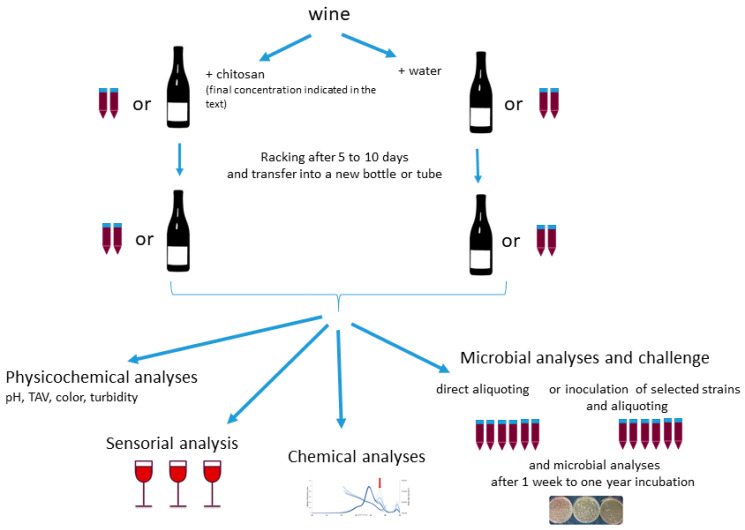
Overview of the experiments performed. Depending on the volume required for further analysis, chitosan treatment and wine storage were performed into bottles or tubes (see specifications in the text).

In order to answer these questions, wines were treated with distinct doses of chitosan and sensorial, chemical, and microbiological analyses were performed. An overview of the experimental design is presented in Figure 1.

### 2.1. Microbial Strains

Three different strains of *B. bruxellensis* were used in this study. According to previous work [21], *B. bruxellensis* strain CRBO L0424 was shown to be sensitive to chitosan, while *B. bruxellensis* strain AWRI 1608 and CRBO L14183 were clearly shown to be tolerant. Before launching the assays, strains were gradually adapted to each studied wine, according to Paulin et al. [21]. When necessary, implantation controls were performed by microsatellite analysis according to [29].

### 2.2. Wines and Culture Media Used

Several wines were used for all these experiments (described in the Supplementary Materials S1–S3). The HW, JW, CW, A, B, C, D, and commercial wine are red wines while wines E and F are white ones.

Total yeasts (TY), non-*Saccharomyces* yeasts (NSY), lactic acid bacteria (LAB), and acetic acid bacteria (AB) counts were performed by plating on selective media after serial dilutions as described by Miot-Sertier et al. [22].

### 2.3. Chitosan

A fungal chitosan batch named F1 and sourced by BioLaffort (Floirac, France) was used. It perfectly fulfilled the oenological codex requirements (Oenological Codex 2023 Edition). It displayed a mean molar weight (MW) of 32 kDa and an acetylation degree (DA) of 9.6% [21].

### 2.4. Wine Chemical Composition Analysis

#### 2.4.1. Wine Treatment with Chitosan Before Analysis

The wine was distributed into 8 tubes (13 mL/tube). A 100X aqueous fungal chitosan suspension (BioLaffort) was diluted into the two first tubes to reach a final concentration of either 40 mg/L, (i.e., 4 g·hL^−1^). The same was performed for the two following tubes to reach a final concentration of 100 mg/L (i.e., 10 g·hL^−1^), and for the two last to reach a concentration of 250 mg/L (25 g·hL^−1^). Control tubes were prepared adding the same water volume as chitosan suspension. After gentle homogenization, wines were left to settle at 16 °C. After 10 days, wines were racked. The first 12 mL of each tube was gently removed, and transferred into a new tube. This constituted the “racked wine”, while the remaining part was thrown out. Two distinct analytical methods were then used to examine the wine composition.

#### 2.4.2. Turbidity, Color and Other Wine Parameter Analysis

The turbidity of the wines prepared for sensorial analysis was assessed with a nephelometer (AQ3010 Thermo Scientific™, Illkirch, France). Results were expressed in nephelometric turbidity units (NTUs).

The CIELab scoring was used to evaluate wine color, with the lightness (from black to white, L*), the color (from blue to yellow, b*), the color (from red to green, a*), the chroma or saturation (C*), and the hue angle (H*), using the method OIV-MA-AS2-11: determination of chromatic characteristics according to CIELab. The color difference was calculated using the CIELab formula (∆E*_ab_).

The total acidity, volatile acidity, pH, alcohol, and total and free SO_2_ contents of the wines prepared for sensorial analysis were evaluated according to the methods recommended by the International Organization of Vine and Wine (OIV). Glucose and fructose concentrations were evaluated by enzymatic method using Megazyme^®^ D-Fructose/D-Glucose test kit (Ayr, UK).

#### 2.4.3. Isolation of Polysaccharide (PS) and Oligosaccharide Fractions and Analysis

The total wine carbohydrate fractions were isolated as previously described [27]. Briefly, wines (5 mL) were depigmented in polyamide CC6 columns (Macherey-Nagel™, Hoerdt, France) previously equilibrated with NaCl 1 M (Thermo Fisher Scientific™, Illkirch, France). Wine polysaccharides (PSs) and oligosaccharides not retained in polyamide column were eluted by two bed volumes of 1 M NaCl. High-performance size-exclusion chromatography (HPSEC) was performed by loading 2 mL of the concentrated total wine carbohydrate on a Superdex-30 HR column (60 × 1.6 cm, Pharmacia, Sweden) with a precolumn (0.6 × 4 cm), equilibrated at 1 mL min^−1^ with 30 mM ammonium formate with a pH of 5.6. The elution of polysaccharides and oligosaccharides was monitored with an Erma-ERC 7512 (Erma, Tokyo, Japan) refractive index detector. The isolated fractions were freeze-dried, re-dissolved in water, and freeze-dried again four times to remove the ammonium salt.

The neutral and acidic sugar molar compositions were determined by GC of the per-O-trimethylsilylated methyl glycoside derivatives after solvolysis with anhydrous MeOH containing 0.5 M HCl (80 °C, 16 h) followed by N-re-acetylation of osamines by addition of acetic anhydride [30,31].

Molar mass distribution, molar weight, and number-average mass (MW and Mn in g·mol^−1^), polydispersity index (MW/Mn), and intrinsic viscosity ([ŋ] in mL g^−1^) were determined at 25 °C, as described by Assunção Bicca et al. [32]. In brief, the determination device combined size exclusion chromatography with a multiangle light scattering (MALS), a differential viscometer, and a differential refractive index detector. SEC elution was performed on a OH-pack guard followed by two serial Shodex OH-pack KB-804 and KB805 columns (0.8 × 30 cm; Shodex Showa Denkko, Japan). The eluant (1 mL min^−1^ flow rate) consisted of 0.1 M LiNO_3_ (filtrated on a 0.1 µm filter unit). The MALS photometer, a DAWN-HELEOS from Wyatt Technology Inc. (Wyatt Technology Corporation, Santa Barbara, CA, USA), was equipped with a GA–AS laser (λ = 658 nm). The differential viscometer detector (Viscostar II, Wyatt Technology Inc., USA) was equipped with a 4-capillary bridge design. The concentration of each eluted polysaccharide was determined using the differential refractive index detector (Optilab TrEX, Wyatt Technology Inc., USA). For molar-mass estimation, all collected data were analyzed using Astra V 6.0.6 software with the Zimm plot (order 1) technique and a differential refractive index increment. A dn/dc classical value was employed for polysaccharides (0.146 mL g^−1^) [33].

#### 2.4.4. Untargeted Analyses of Molecules Between 180 and 1800 g/mol by LC-HRMS

Analyses were performed on a binary Vanquish Flex ultra-high performance liquid chromatography (UHPLC) chain, coupled with a VWD Vanquish Flex UV/VIS detector and a high-resolution mass spectrometer (HRMS) Q-Exactive PLUS HCD equipped with a HESI-II (heated electrospray ionization) (Thermo Fisher Scientific™) source. The Accucore RP-MS column (100 mm × 2.1 mm, grain size 2.6 μm), equipped with an Accucore RP-MS pre-column, was held at 30 °C during the analyses.

Chromatographic analysis was performed in gradient mode at 0.3 mL/min with Milli-Q^®^ water (solvent A) and acetonitrile (grade LC-MS, Sigma Aldrich^®,^ L’isle d’Abeau, France) (solvent B), both acidified with 0.1% formic acid (Thermo Fisher Scientific™). The gradient moved from 2 to 50% B in 15 min, 50 to 100% B between 15 and 17 min, and kept at 100% B until 20 min.

The scans were performed using Xcalibur software (version 4.1.31.9, Thermo Fisher Scientific™) and the mass spectrometer was controlled with the device’s Q Exactive HF Tune program (version 2.12).

UV detection was performed at the following four wavelengths: 230, 280, 306, and 325 nm. The samples were analyzed in full scan mode with the first method, while the second method used the ddMS^2^ (data-dependent MS-MS) mode.

Retention times and *m/z* ratios were rounded to 4 and 2 digits, respectively, and a R script on RStudio software (version 1.4.1717, RStudio Team, Boston, MA, USA) was used to eliminate duplicates: this was allowed to go from 4552 to 224 ions.

### 2.5. Long Lasting Antimicrobial Impact

Assay in HW wine. The wines were introduced into three 2 L bottles. Ten milliliters of an aqueous suspension containing 200 mg of chitosan was added to the first bottle (i.e., 10 g·hL^−1^). The second bottle received 10 mL of water. The treated and control wines were mixed by bottle inversion and stored at 16 °C for 5 to 10 days depending on the assay. Then, the treated and untreated wines were racked (the volume left was about 140 mL) and transferred to new 0.75 L bottles and a series of 10 mL tubes. After a rest of 0, 1, 2, and 13 weeks, two tubes of each modality of treatment (i.e., 6 tubes) were removed, analyzed for microbial population, and then one of the tubes of each modality was inoculated with *B. bruxellensis* CRBO L0424. The tubes were then transferred at 20 °C, and monitored for microbial population over 4 to 13 weeks. After a rest of 52 weeks post-racking, tubes containing the control and tubes containing the 10 g·hL^−1^-treated wine were inoculated with either *B. bruxellensis* (CRBO L02424, CRBO L14184 or AWRI 1608, 1 strain by tube) or nothing (control). The wines were then analyzed for microbial population just after inoculation and after a 1 week incubation at 20 °C.

Assay in CW and NW wines. The wines were introduced into three 2 L bottles. Ten milliliters of an aqueous suspension containing 80 mg of chitosan was added to the first bottle (i.e., 4 g·hL^−1^). The second bottle received 10 mL of an aqueous suspension containing 200 mg of chitosan (i.e., 10 g·hL^−1^), and the third one received 10 mL of water. The treatment was performed as in the HW wine and the racked wines transferred into as many tubes as necessary, and left to stand at 16 °C for two weeks after racking. Then, five tubes of each modality were inoculated with strain CRBO L14183, five with strain AWRI 1608, five with strain CRBO L0424, and 5 others were used as controls (un-inoculated). At each sampling time, a tube was removed from the device, and analyzed for microbial population.

### 2.6. Sensory Analysis

The wine was introduced into 2 magnum bottles (1.5 L/bottle). Five milliliters of an aqueous suspension containing 150 mg of chitosan was added to the first bottle (final concentration 10 g·hL^−1^). The second bottle received 5 mL of water. The headspace was inerted with CO_2_. Each bottle was mixed by inversion. Both bottles were stored at 16 °C for 10 days. Then, the treated and untreated wines were racked (the volume left was about 140 mL) and transferred to new magnum bottles. Sterile beads were added to fill the bottles and reduce headspace, then inerted with CO_2_. The wines were stored overnight at 16 °C. Two hours before tasting, the samples were placed at room temperature, away from light and heat sources. Fifteen minutes before tasting, wines were served in glasses identified with three-digit numbers. Each glass was covered with a mobile cap to minimize the loss of volatile compounds.

#### 2.6.1. Tasting Conditions

Sensory analysis was performed in a dedicated room, at room temperature (around 20 °C) (ISO 8589:2007 [34]) and with INAO-normalized black glasses (ISO 3591:1977 [35]). All sensory procedures performed in this study involving human participants were in accordance with the Helsinki Declaration or comparable ethical standards. Twenty-nine voluntary subjects (students and employees of ISVV Institute, 54% women; aged between 24 and 51 years with a mean of 30.5 ± 7.9 years) were recruited. Participants gave informed written consent prior to tasting. In particular, they were informed that the evaluated beverages were commercial red and white wines potentially supplemented with food grade oenological products. They were asked to taste, grade, and spit out of these samples. They were also informed about the safety and confidentiality of this analysis and their ability to participate or withdraw from it at any time. A more specific ethical permission to conduct a human sensory study was not a requirement of the institution where this study was conducted. The evaluations were carried out as a single run for at most two distinct wines, and one run in a day, between 10:00 and 12:00 a.m. For each panelist, each session took about 10 min.

#### 2.6.2. Sensory Tests

The sensory impact of chitosan was evaluated by psychophysical tests. Triangle tests (ISO 4120:2021 [36]) were implemented with black glasses to investigate whether the treatment with chitosan modifies the olfactory, gustatory, or tactile properties of the wines.

The panelists were presented with three samples simultaneously (two identical), and asked to identify which sample was different, considering all the sensory characteristics, encompassing smell, taste, flavor, and body. The presentation sequences of the samples were randomized, judge by judge. Even though commercial wines were used and chitosan was food quality grade, the panelists were advised to spit out the samples after tasting.

### 2.7. Statistical Analysis

The results were processed as recommended by ISO for triangular tests (ISO 4120:2021) by using a binomial law; *p* values lower than 0.05 were considered for significant results. Multivariate analysis was performed with the packages ade4 (R package version 1.7.18, RStudio Team, Boston, MA, USA) and ggpubr (R package version 0.4.0).

## 3. Results and Discussion

### 3.1. Chemical Composition Analysis of Chitosan Treated Wines

Two wines (HW and a commercial wine) were treated by chitosan (0, 4, 10, and 25 g·hL^−1^) and analyzed. The molar percentages of the main monosaccharides released after solvolysis of wine polysaccharides and oligosaccharides were determined by GC analysis of their per-O-trimethylsilylated methyl glycoside derivatives. The results are presented in Table 1.

Galactose and galacturonic acid originating from grape berry cell walls, mannose constituting yeast mannoproteins, and glucose originating either from grape cell wall hemicelluloses or bacterial polysaccharides were the main monosaccharides found in both HW wine and the tested commercial wine. Arabinose, rhamnose, and xylose were also present at smaller percentages, primarily in the oligosaccharide fraction of the wines. The main monosaccharides released from the glycosides present in the controls and in the chitosan-treated wines were thus similar to those already identified in other wines, as reported previously [37]

To identify and quantify the residues of N-acetyl-glucosamine left by chitosan treatment at various doses (0, 4, 10, and 25 g·hL^−1^), purified fractions of polysaccharides and oligosaccharides were N-acetylated after methanolysis and before trimethylsilylation [30]. After derivation of the polysaccharide fractions, no residue of N-acetyl glucosamine could be identified or detected in the HW or in the commercial wine that received a dose of chitosan of 4 g·hL^−1^ or 10 g·hL^−1^ (oenological dose). The results were similar for the wines that received an overdose of chitosan (25 g·hL^−1^, Table 1). However, in the purified oligosaccharide fraction of the commercial wine, residues of N-acetylglucosamine were detected. A dose-dependent increase in the N-acetylglucosamine proportion was noticed when comparing the results of the control with those of the 10 g·hL^−1^ or 25 g·hL^−1^-treated wines (+1 to 1.5%). This seems to indicate that very small soluble chitosan oligosaccharides may persist in the wines after treatment with high doses (10 and 25 g·hL^−1^). However, this phenomenon was not observed in the oligosaccharide fraction of the HW-treated wine (Table 1).

Static (MW, Mn, Rg) and dynamic ([η], Rh) average molecular parameters observed in HPSEC-MALS profiles of the polysaccharide fractions of HW and commercial wines treated with 0, 4, 10, and 25 g·hL^−1^ of chitosan F1 are provided in Table 2 (see next page).

The predominant populations in the polysaccharide fractions of wine HW displayed an average MW of 1.312 × 10^5^ g·mol^−1^ and a polydispersity index of 2.21. For the commercial wine, an average MW of 1.03 × 10^5^ g·mol^−1^ and a polydispersity index of 4.05 were obtained. Purified polysaccharides from commercial wine had a lower average molecular weight (MW) but a much higher polydispersity than those in the HW wine. The addition of chitosan at doses of 4, 10, and 25 g·hL^−1^ did not have a significant effect on the static (MW, Mn, Rg) and dynamic ([η], Rh) parameters of the polysaccharides observed by HPSEC-MALS for the two wines considered.

Regarding the polysaccharide molar-mass distribution, six delimited ranges were considered (molar mass range: range 1 = 2.5–20 × 10^3^ g·mol^−1^; range 2 = 20–100 × 10^3^ g·mol^−1^; range 3 = 100–250 × 10^3^ g·mol^−1^; range 4 = 250–500 × 10^3^ g·mol^−1^; range 5 = 0.5–1 × 10^6^ g·mol^−1^; range 6 = 1–10 × 10^6^ g·mol^−1^). F1 chitosan mainly contains molecules in range 2, with a small proportion in range 1. In HW wine, the low-mass fraction (range 1) increased when the highest doses of chitosan were added to the wine (10 and 25 g·hL^−1^), while for commercial wine, it decreased (Table 2). However, in the same time, the fraction corresponding to range 2 decreased in HW wine, while it increased in commercial wine. Since chitosan was not present in the treated wine polysaccharides (no significant N-acetylglucosamine content detected in the polysaccharide fraction, see above), these changes may be linked to the chitosan-induced precipitation of intermediate-mass macromolecules. It could be also explained by a chitosan-induced breaking up of high molecular weight polysaccharides aggregates, leading to a decrease in the apparent average molecular size [38].

However, no consensus effect of chitosan treatment on the chemical composition of the wine could be evidenced using this analytical method of wine chemical composition.

A third type of analysis was then considered. Six wines (four reds and two whites) treated with 0, 4, 10, and 25 g·hL^−1^ were analyzed by UHPLC-ESI(-) HRMS (cut-off threshold 180 to 1800 g·mol^−1^ approx.). This method was expected to make visible either the ionizable chitosan oligosaccharides left in the wine after treatment, racking, and filtration (0.45 µm cut off), or the small elements removed from the wine due to aggregation and precipitation with chitosan. The treatment “overdose” of 25 g·hL^−1^ was used again in order to maximize the effects of the treatment, if any.

**Figure 2:**
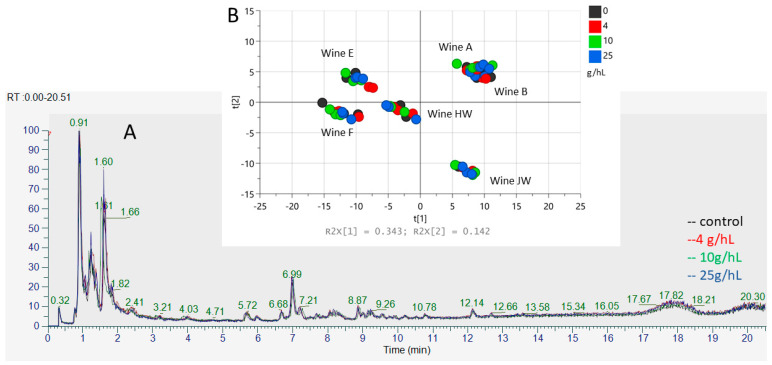
(**A**). UHPLC chromatograms obtained for treated and untreated HW wine. (**B**). PCA analysis on dataset of 224 ions analyzed by UHPLC-ESI(-) HRMS of 6 treated and untreated wines. Three doses of treatment and three treatment replicates were considered.

The treatment induced a dose-dependent decrease in sample filterability of red wines B and JW, but not for the other wines examined. The chromatograms and the dataset extracted from the HRMS device showed no visible dose-dependent and reproducible difference. Examples of chromatograms obtained are presented in Figure 2A.

The dataset of 224 ions was then examined and a PCA of wine composition was realized (Figure 2B). Spots corresponding to the treated and untreated samples for the same wine appeared grouped. In each group (i.e., for each given wine), treated and untreated samples appeared distributed independently from the treatment dose. Individual PCAs made on each treated wine are presented in the Supplementary Materials S1–S3. In the same way, the distribution of the spots on the individual PCA was not associated to the chitosan dose used. This non-targeted method did not allow the identification of any signal directly associated with chitosan treatment.

Chitosan preparations that meet the requirements of the Oenological Codex are more than 95% insoluble [10]. Wines treated with 25 g·hL^−1^ should not contain more than 12.5 mg/L of chitosan after racking, which could represent from 3 to 20% of the oligo and PS present [37]. However, none of the analytical methods employed and described in Section 3.1. has identified a signal systemically associated with chitosan treatment even if some slight changes were noticed in the wine oligosaccharide and polysaccharide fractions. In other wines, Arenas et al. [39] noted a sharp decrease in PS content in treated white wines but higher chitosan doses (100 g·hL^−1^) were used. Filipe Ribeiro et al. [40] reported a dose-dependent change in phenolic compounds content, but they examined a single wine. Indeed, we also noticed slight composition changes but these changes differed from one wine to the other and it is therefore impossible to identify a treated wine without comparing a treated wine with its untreated control. The variability in the chemical composition of the wines may induce a variability in what aggregates with chitosan and what remains in the wine, either chitosan or wine molecules. The wine pH may also modulate these phenomena [14]. However, the wines examined in this study displayed quite close pH values (from 3.3 to 3.8) which suggests that other parameters may be responsible for the variations observed.

### 3.2. Impact of Treatment on Oenological Parameters and Organoleptic Properties

Six wines (A to F), four reds and two whites, were treated at the maximum dose allowed by the Eonological Codex for antiseptic purpose (10 g·hL^−1^). At t0, the six wines displayed low microbial populations (<10^3^ CFU·mL^−1^). Yeasts and bacteria populations (TY, NSY, LAB and AB) enumerated at day 10 in the treated and untreated wines remained low; i.e., no significant microbial development or death occurred in untreated wine compared with its treated counterpart or conversely. Wines were also analyzed for different oenological parameters (glucose + fructose concentration, SO_2_, alcohol by volume (ABV), total acidity, pH, volatile acidity). No significant differences were observed (Supplementary Materials data S1–S3).

**Figure 3:**
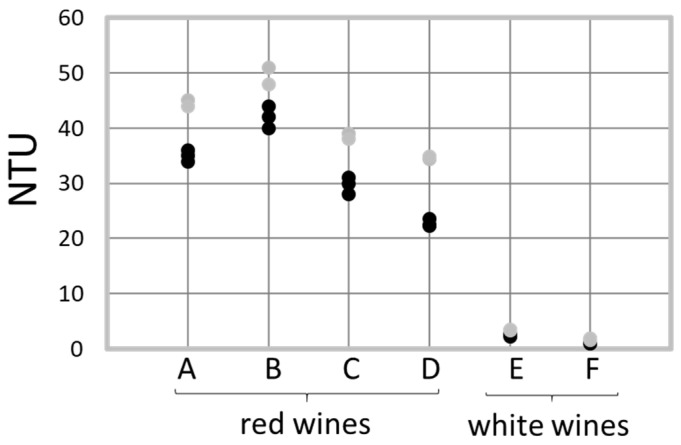
Turbidity analysis of untreated (black) and treated (gray) wines. For all the wines examined, the differences between treated and untreated were significant- (*p* value < 0.05, 3 assays), but it was not perceived with the naked eye, except for wine E.

Then, the turbidity of wines was analyzed. In all assays, a significant (*p* < 0.05) turbidity increase induced by treatment was observed (Figure 3). However, the overall turbidity remained in the order of magnitude generally reported for each class of wine. Indeed, in white ones, the overall turbidity remained very low (<5 NTU). Differences were not noticeable to the naked eye in wine F, but they were in white wine E. Chitosan therefore certainly induces a particle aggregation that leads to greater turbidity, as previously reported for other food solutions [11,13,18]. In red wines, the initial turbidity was higher and changes induced by chitosan treatment were hardly perceptible without specific devices. Furthermore, turbidity measurement did not enable to identify a treated wine without comparison with the untreated control, because a treated wine could display turbidity lower than that observed in other untreated wines. This low-dose effect of chitosan treatment is quite surprising because at higher doses, chitosan is recommended for cloudiness prevention or treatment [13]. Nevertheless, Strand et al. [35] indicated that cloudiness prevention works better using chitosans with MW larger and DA higher than those recommended for usual enological chitosan, such as the F1 used in this study.

The color of the wine was also studied. No change was noticeable to the naked eye and CIELab measures were only slightly modified by chitosan treatment (E*ab < 1) in five out of six cases of the examined wines A to F. Only wine A displayed a color difference slightly higher than this E*ab threshold value (Table 3), but far below 3 and not noticeable to the naked eye by most observers.

In order not to influence the tasters who would be able to perceive the turbidity changes, the sensory properties of treated and untreated wines were analyzed in black glasses. The results of the triangular tests are shown in Table 3. None of the treated wines was significantly differentiated by the panel. This also applied to wines A and E, which displayed slight differences in color or turbidity, respectively, after treatment. Moreover, when some panelists designated the treated wine as different, they generally described it as less aromatic or less oxidized than the control, but there was no consensus that one of the modalities was better than the other.

As for chemical analyses, none of the analyses presented in Section 3.2 and performed in our laboratory made it possible to establish a criterion that enabled the recognition, for sure, of a treated wine among several wines, without comparing with the untreated control. Ferrer-Gallego et al. [40] reported more important color changes on Albariño and Tempranillo varietal unspoiled wines, suggesting that chitosan may interact with anthocyans and other phenolic compounds. However, these were not associated with a significant change in organoleptic appreciation of the wine. On the opposite, in spoiled wines, Filipe Ribeiro et al. [41] reported an improvement in the sensorial perception after treatment, while Colangelo et al. [42] reported a reduction in free terpenols. At higher doses and a much longer time of contact (one year, overdose of 25 g·hL^−1^), Castro Marin et al. [13] reported higher values of wine fruity character in the treated wines. However, this sensorial difference was not perceived after one single month of contact.

### 3.3. Long Term Consequences of Chitosan Treatment on Microbial Development

Previous work has shown that chitosan-treated wines sometimes displayed delayed malolactic fermentation [22]. This suggests that the treatment leaves a trace (either chitosan residues or essential nutrients depletion), which affects microorganisms already present in the wine or inoculated after treatment. As the main target of the treatment being *B. bruxellensis*, we examined whether the treated wines were protected in a persistent way, in particular in case of subsequent contamination.

**Figure 4:**
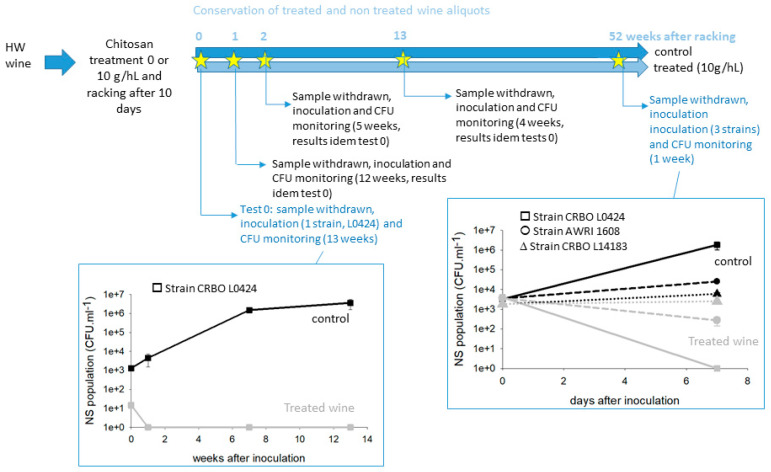
Long-term microbial stability induced on HW wine by chitosan treatment. The wine was treated with distinct chitosan doses and stored several weeks at low temperature. At the indicated time, a portion of treated and untreated wine was inoculated with distinct strains and incubated for several weeks at 20 °C. *B. bruxellensis* cultivable population kinetics are indicated for experiments performed after 0 and 52 weeks storage.

Two separate experiments were conducted in three different wines. The first one was conducted with HW wine. Before starting, the HW wine was pasteurized. It did not contain cultivable microorganisms anymore and it was treated with two doses of chitosan (4 and 10 g·hL^−1^) or with water (mode 0), racked, and stored for 52 weeks. At 0, 1, 2, 13, and 52 weeks of storage after racking, the cultivable NS populations of the treated and untreated wines were analyzed: as expected, none displayed any detectable population. The wines collected after 0, 1, 2, 13, and 52 weeks of storage were then inoculated with different strains of *B. bruxellensis*: a chitosan-sensitive strain (CRBO L0424) for the first trial, and this sensitive strain plus two tolerant strains (AWRI 1608 and CRBO L14183) for tests performed after 52 weeks of storage. Figure 4 summarizes the experimental protocol and presents the growth kinetics observed after inoculation at 0 and 52 weeks of storage.

Regardless of the “age” of the treated wine, the sensitive strain never survived even a week after inoculation in the treated wines, while it always did in the untreated stored wine. This suggests that the treatment with chitosan leaves a trace in the wine, a trace detrimental to the sensitive yeast. This trace was still present one year after treatment. On the opposite, tolerant strains survived during the first week after inoculation in the treated wine stored one year, despite a slight loss of cultivability in the case of strain AWRI 1608 (Figure 4). Indeed, strain *B. bruxellensis* AWRI 1608 population was divided by 1.33 in a week in the treated wine, while, in the same time, it was multiplied by 2.8 in the untreated control. The strain *B. bruxellensis* CRBO L14183 behaved the same in treated and untreated wines stored 52 weeks before inoculation. This experiment suggests that chitosan traces still affect the microbial development one year after treatment. However, all *B. bruxellensis* strains are not affected the same way.

**Figure 5:**
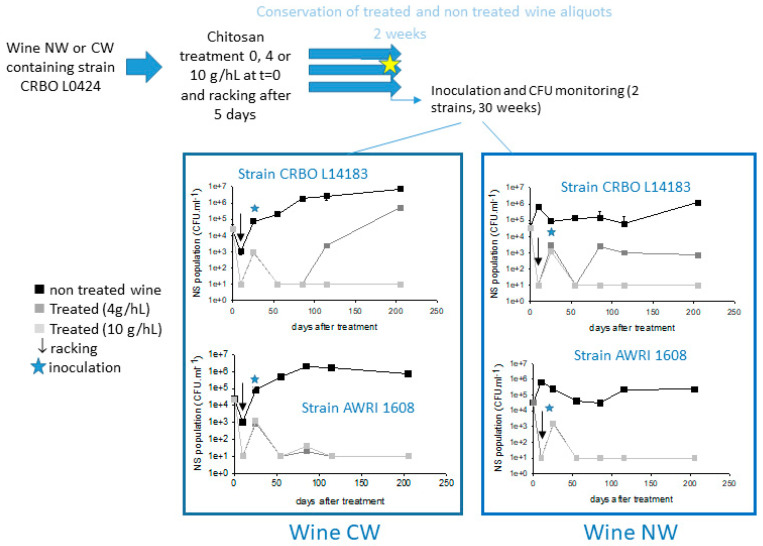
Long-term stability induced on CW or NW wines by chitosan treatment. The wines were inoculated at t = 0 with *B. bruxellensis* CRBO L0424 (sensitive) and then treated with distinct chitosan doses, racked on day 5, stored 15 days at low temperature. At the time indicated by a star, two samples of each treated or untreated wine were separately inoculated with two distinct *B. bruxellensis* tolerant strains (CRBO L14183 and AWRI 1608) and the four inoculated wines were incubated 30 weeks at 20 °C. *B. bruxellensis* cultivable population kinetics are indicated for each treated wine and each strain. The controls treated at t = 0 racked at t = 5 days and un-inoculated at t = 20 days are not presented on the figure: no detectable CFU were recovered from these wines over 200 days.

In the second experiment, two distinct wines (CW and NW) contaminated at t = 0 by strain *B. bruxellensis* CRBO L0424 were treated, racked, stored for two weeks, and then inoculated with distinct strains of *B. bruxellensis* (Figure 5).

Unlike the HW wine studied in Figure 4, these two wines contained a strong initial population of *B. bruxellensis* sensitive strain (>10^4^ CFU·mL^−1^). In the two control wines that did not receive chitosan, the yeast population dynamic was different during the 10 days before racking; the population decreased from 10^4^ to 10^3^ in CW wine, while it increased from 10^4^ to 10^6^ in NW wine. Meanwhile, in the treated wines (4 or 10 g·hL^−1^), populations fell to levels below the detection threshold after racking. In the absence of subsequent inoculation, the population remained at this undetectable level over the next 200 days.

Fifteen days after racking (t = 20 days), part of the treated and untreated wines were inoculated with two distinct *B. bruxellensis* tolerant strains, AWRI 1608 and CRBO L14183. In untreated wines, the strains initially present as well as the added strains maintained their population levels or even developed. Implantation control were carried out throughout the experiment to verify and show the presence of both strains.

In the treated wines, the population rebound at t = 20 days reflected the cultivability of the strains inoculated at that time (Figure 5). Nevertheless, regardless of the wine and the chitosan dose used for initial wine treatment, strain *B. bruxellensis* AWRI 1608 finally became undetectable. The strain *B. bruxellensis* CRBO L14183 also became undetectable in wines treated at 10 g·hL^−1^. However, when the treatment dose was lowered to 4 g·hL^−1^, the strain CRBO L14183 first disappeared wine but it then developed again.

In CW and NW wines, the chitosan treatment thus eliminated the *B. bruxellensis* sensitive strain initially present and in certain cases, it also prevented the development of tolerant strains which contaminated the wine after treatment. The treatment with chitosan would therefore present a long-lasting inhibitory effect against *B. bruxellensis* development; however, the long-lasting protection depends on the chitosan dose used and on the strains which contaminate the wine.

The experiments presented in Section 3.3 all suggest that the chitosan treatment also leaves residues in the treated wine. Since no chemical trace of chitosan residues was detected in wine HW (Section 3.1), the residual effects could be due to other elements not quantifiable or detectable by the method used. The remanence may also be linked to elements eliminated by chitosan treatment. Several studies mention that chitosan treatment may lead to the sequestration of potassium, iron, calcium, and divalent ions [27,42,43], or to a significant decrease in available nitrogen [44]. Colangelo et al. [42] also reported a reduction in tartaric acid and malic acid. Strand et al. [42] indicated that salts and, in particular, NaCl, modulated chitosan-induced flocculation. Aggregation and sedimentation of phenolic compounds or nutrients may also occur in the presence of chitosan, as chitosan is sometimes used to extract phenolics from fruit preparations [45]. However, (1) the target compounds are not quantified by the different methods used here or (2) many compounds are affected in a very small proportion, which makes the changes undetectable.

Although treatment residues cannot be excluded, the training of salts and other charged elements could explain the long-term protection observed for HW-, CW-, and NW-treated wines. Several hypotheses can be proposed, as follows: (1) The chitosan treatment eliminated elements that could be essential for yeast growth or even survival. As *B. bruxellensis* is a yeast species with a very high genetic and phenotypic diversity, including as concerns nutritional requirements or sensitivity to phenolic compounds [46], the impact of limiting elements may be variable depending on the strain considered. (2) *B. bruxellensis* cell membranes display different Zeta potential depending on the genetic group [47]. Apparent phospholipids or different membrane fluidities may modify cell interaction with chitosan and microbial strain sensitivity and this could explain the diverse sensitivities to primary treatment [21,43]. The salts or ionic elements present in the untreated wines may either mask the charged residues present on the cell surface or compete with the cell for interaction with chitosan. Their depletion may enable the direct action of chitosan on the target cells, which could explains why very low (undetectable) level of residue remain active.

## 4. Conclusions

Fungal chitosan treatment is not perceptible in a sure and systematic way, neither through the chemical analyses proposed in this article, nor by sensory analysis (tasting, observation with the naked eye), nor by analysis of classical oenological parameters. This means that as mentioned in other studies, chitosan treatment has no adverse effect on wines used in this study. Nevertheless, whatever the wine examined in this study, the antiseptic effect is strongly persistent on sensitive strains, whether these strains are present in the wine before treatment or inoculated after. For tolerant strains, the treatment is less effective and less durable; the residual effects are less strong.

Further work will be necessary to identify the exact target of chitosan in wine and/or on microbial surfaces, but we cannot exclude that many exist and combine their effects, as already suggested [14]. Once the targets are identified, it would be interesting to explain why treatment efficiency varies depending on the wine and on the strain [21] and to propose models to predict the short- and long-term consequences on sensorial quality and aroma complexity of the treated wines.

## Figures and Tables

**Table 1:** Molar glycosyl-residue composition determined by gas chromatography coupled to a mass spectrometer (GC-MS) analysis of their per-O-trimethylsilylated methyl glycoside derivatives after solvolysis of polysaccharides and oligosaccharides of HW and commercial wines treated with 0, 4, 10, and 25 g·hL^−1^ of chitosan F1.

			Wine HW				Commercial Wine			
		**Chitosan F1**	**0 g·hL^−1^**	**4 g·hL^−1^**	**10 g·hL^−1^**	**25 g·hL^−1^**	**0 g·hL^−1^**	**4 g·hL^−1^**	**10 g·hL^−1^**	**25 g·hL^−1^**
Polysaccharide fractions	Molar %	Ara	-	2.0	3.4	2.4	2.7	9.3	4.1	4.5
		Rha	-	-	-	-	1.5	3.6	1.5	1.3
		Fuc	1.0	-	-	-	0.7	1.3	1.0	0.4
		Gal	33.8	47.8	44.4	49.9	17.7	18.3	29.1	24.4
		Glc	19.3	16.4	16.5	16.4	9.5	4.4	5.5	3.2
		Man	16.8	22.6	26.5	25.9	39.2	39.5	41.4	47.8
		GalA	26.9	8.6	5.8	3.8	22.1	18.7	13.4	13.7
		GlcA	2.2	2.6	2.7	1.7	5.2	3.9	3.2	2.4
		GlcNAc	-	-	-	-	-	-	-	-
Oligosaccharide fractions	Molar %	Ara	1.8	3.2	2.6	1.5	9.6	10.8	11.5	6.7
		Rha	0.2	0.4	0.2	0,0	6.7	4.6	8.5	4.5
		Fuc	0.3	0.4	0.4	0,0	1.0	0.7	1.1	0.8
		Gal	6.1	5.8	6.7	5.4	15.8	39.2	17.4	20.0
		Glc	15.9	15.1	14.8	15.6	17.5	9.2	15.5	18.8
		Man	7.8	7.8	8.0	8.1	12.2	5.8	10.3	13,0
		Xyl	4.3	5.1	4.2	3.8	6.4	3.5	5.6	4.5
		Xyl-ol	0.7	0.7	1.2	0.9	-	-	-	-
		GalA	58.9	57.8	58.2	59.8	23.0	11.3	22.3	24,0
		GlcA	1.5	1.1	1.1	1.4	2.5	11.9	2.8	2.3
		4-O-Me GlcA	2.5	2.7	2.5	3.5	1.9	1.6	1.9	1.9
		GlcNAc	-	-	-	-	1.9	1.6	3,0	3.6

Ara: arabinose; Rha: rhamnose; Fuc: fucose; Xyl: xylose; Xyl-ol: xylitol; Man: mannose; Gal: galactose; Glc: glucose; GlcNAc: N-acetylglucosamine; GalA: Galacturonic Acide; GlcA: Glucuronic Acid; 4-O-Me GlcA: 4-O-methyl-Glucuronic acid. For all assays, the standard deviation was below 5%.

**Table 2:** Average molecular parameters observed in the HPSEC-MALS profiles of the polysaccharide fractions of HW and commercial wines treated with 0, 4, 10, and 25 g·hL^−1^ of chitosan F1. MW: molecular weight in mass, Mn: molecular weight in number, PI: polydispersity index, Rz: radius of gyration, Rh: hydrodynamic radius, [η]: intrinsic viscosity.

		Wine HW				Commercial Wine			
	**Chitosan F1**	**0 g·hL^−1^**	**4 g·hL^−1^**	**10 g·hL^−1^**	**25 g·hL^−1^**	**0 g·hL^−1^**	**4 g·hL^−1^**	**10 g·hL^−1^**	**25 g·hL^−1^**
	Peak Limits (min)	14.460 − 20.144	14.228 − 20.146	14.367 − 20.272	14.535 − 20.249	14.179 − 20.128	14.044 − 20.060	14.038 − 20.133	14.254 − 20.242
**Hydrodynamic radius moments (nm)**	Rh(v)w	6.351 (±0.583%)	6.455 (±0.677%)	6.680 (±0.649%)	6.402 (±0.451%)	5.238 (±0.427%)	5.176 (±0.422%)	5.416 (±0.433%)	5.814 (±0.452%)
**Molar mass (g/mol)**	Mn	5.601 × 10^4^ (±1.022%)	6.122 × 10^4^ (±0.673%)	6.248 × 10^4^ (±0.864%)	5.792 × 10^4^ (±0.920%)	2.346 × 10^4^ (±0.357%)	2.247 × 10^4^ (±0.466%)	2.476 × 10^4^ (±0.535%)	3.200 × 10^4^ (±0.753%)
	Mw	1.413 × 10^5^ (±0.336%)	1.432 × 10^5^ (±0.180%)	1.267 × 10^5^ (±0.240%)	1.136 × 10^5^ (±0.203%)	9.778 × 10^4^ (±0.096%)	9.625 × 10^4^ (±0.130%)	1.026 × 10^5^ (±0.198%)	1.157 × 10^5^ (±0.164%)
**Polydispersity**	Mw/Mn	2.523 (±1.076%)	2.338 (±0.696%)	2.028 (±0.897%)	1.961 (±0.943%)	4.167 (±0.369%)	4.284 (±0.484%)	4.146 (±0.570%)	3.614 (±0.770%)
**rms radius (nm)**	Rz	20.6 (±0.6%)	19.1 (±0.5%)	15.4 (±0.6%)	11.2 (±0.4%)	15.6 (±0.3%)	15.9 (±0.3%)	16.1 (±0.6%)	16.4 (±0.4%)
**Intrinsic viscosity (mL/g)**	[η]w	15.20 (±1.70%)	15.48 (±2.09%)	18.22 (±2.05%)	17.33 (±1.36%)	14.00 (±1.26%)	13.67 (±1.25%)	14.50 (±1.27%)	14.97 (±1.29%)
**% Range 1**	2.5–20,000 g/mol	0.000	0.000	4.920	8.929	43.166	44.386	41.148	30.406
**% Range 2**	20–100,000 g/mol	58.238	56.245	51.342	50.845	27.152	25.397	27.015	33.412
**% Range 3**	100–250,000 g/mol	28.298	30.206	31.510	30.469	17.776	18.401	19.425	21.961
**% Range 4**	250–500,000 g/mol	8.967	9.461	9.869	8.905	9.763	9.971	10.145	11.669
**% Range 5**	0.5–1,000,000 g/mol	3.300	3.017	2.359	0.852	1.871	1.845	2.267	2.553
**% Range 6**	1–10,000,000 g/mol	1.196	1.071	0.000	0.000	0.273	0.000	0.000	0.000

**Table 3:** Color difference and triangle test results for treated (10 g·hL^−1^) compared to untreated wines A to F.

Wine	ΔE*ab	Correct Responses	*p* Value
A (red)	1.67	12	0.10 n.s.
B (red)	0.14	12	0.13 n.s.
C (red)	1.41	11	0.17 n.s.
D (red)	1.00	12	0.11 n.s.
E (white)	0.19	5	0.68 n.s.
F (white)	0.72	12	0.13 n.s.

n.s.: non significant.

## Data Availability

The original contributions presented in the study are included in the article/Supplementary Materials; further inquiries can be directed to the corresponding author.

## Supplementary Materials

The following supporting information can be downloaded at: https://www.mdpi.com/article/10.3390/foods13213367/s1, Data S1: inventory of the wines used; Data S2: individual PCA analysis; Data S3: Oenological parameters of untreated(black) and chitosan treated(10 g/L, gray) wines A to F.
