# Supplementary material for: Does Fungal Chitosan Leave Noticeable Traces in Treated Wines?
Source: Foods. 2024 Oct 23;13(21):3367. doi: 10.3390/foods13213367 (PMC11544894; doi:10.3390/foods13213367)
Supplement: Supplementary file 1 [file foods-13-03367-s001.zip › foods-3231584-supplementary.pdf]

Supplemental data 1. Inventory of the wines used.

	Wine HW	JW	NW	CW	A	B	C	D	E	F	Commercial Wine
Used in section	3.1, 3.2, 3.3	3.1	3.3	3.3	3.1, 3.2	3.1, 3.2	3.2	3.2	3.1, 3.2	3.1, 3.2	3.1
Grape variety, vintage	Blend from different grape varieties, 2019	CS + Merlot 2023	Merlot, 2020	Merlot, 2020	CS 2020	CS + Merlot 2020	Syrah-Grenache 2020	Bourgogne début élevage, 2019	Sauvignon, muscat petit grain 2020	Sauvignon Sémillon 2020	IGP Pays de l'Hérault Coteaux de Bessilles 2022
pH	3.57	3.60	3.44	3.68	3.65	3.50	3.80	3.40	3.35	3.50	ND
Sugars g/L	ND	0.57	ND	0.20	0.22	0.27	0.34	0.27	0.31	0.19	ND
TAV % vol	12.57	13.3	14.55	14.35	14.00	11.50	14.10	13.92	12.30	12.43	ND
Malic acid g/L	ND		ND	0	ND	ND	ND	ND	ND	ND	ND
Total SO_2 mg/L	ND	ND	14	13	25	90	40	20	84	110	ND

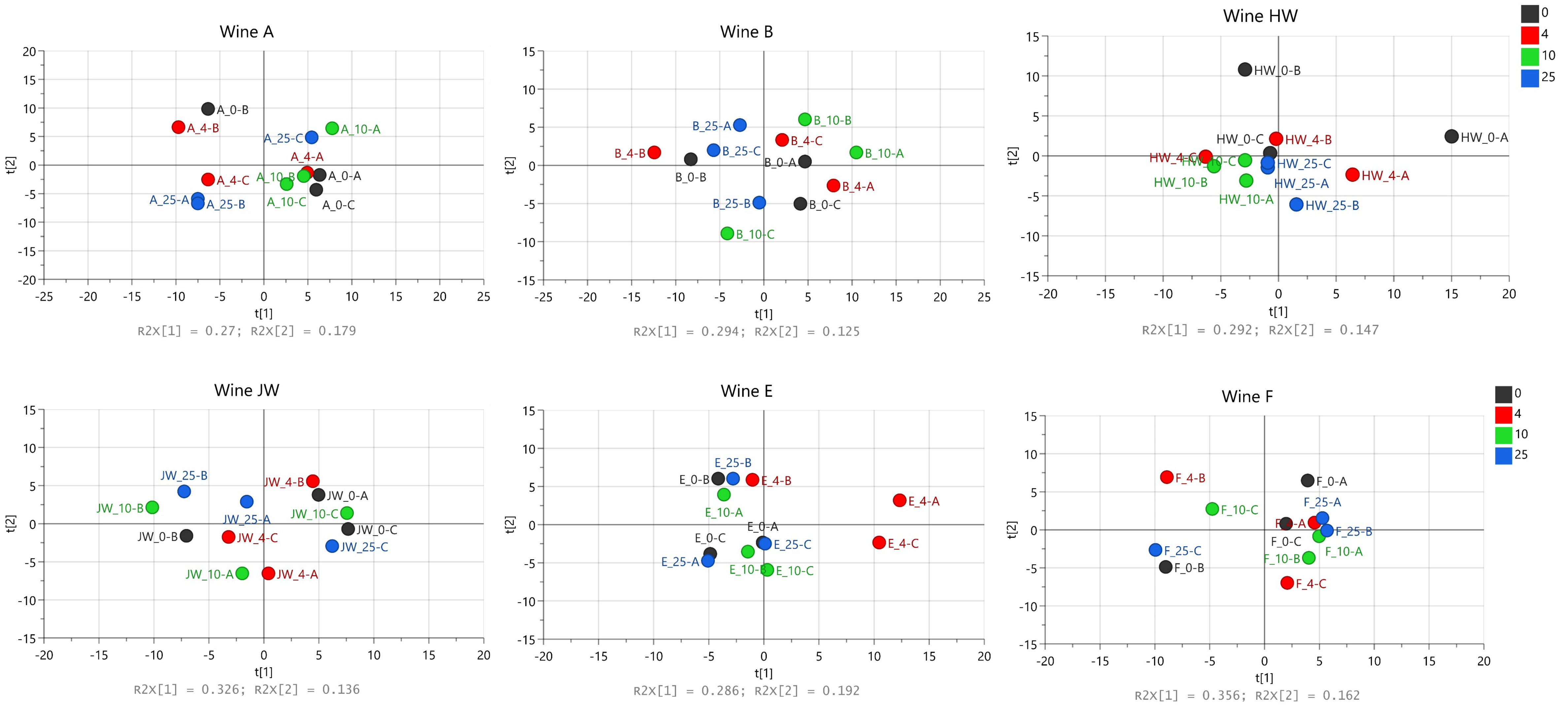

Supplemental data 2. Individual PCA analysis of 6 wines analyzed treated with chitosan and analyzed by UPLC-ESI(-) HRMS. A dataset of 224 ions, four doses of treatment (0, 4, 10 and 25 g/hL) and three replicates for each dose were considered.

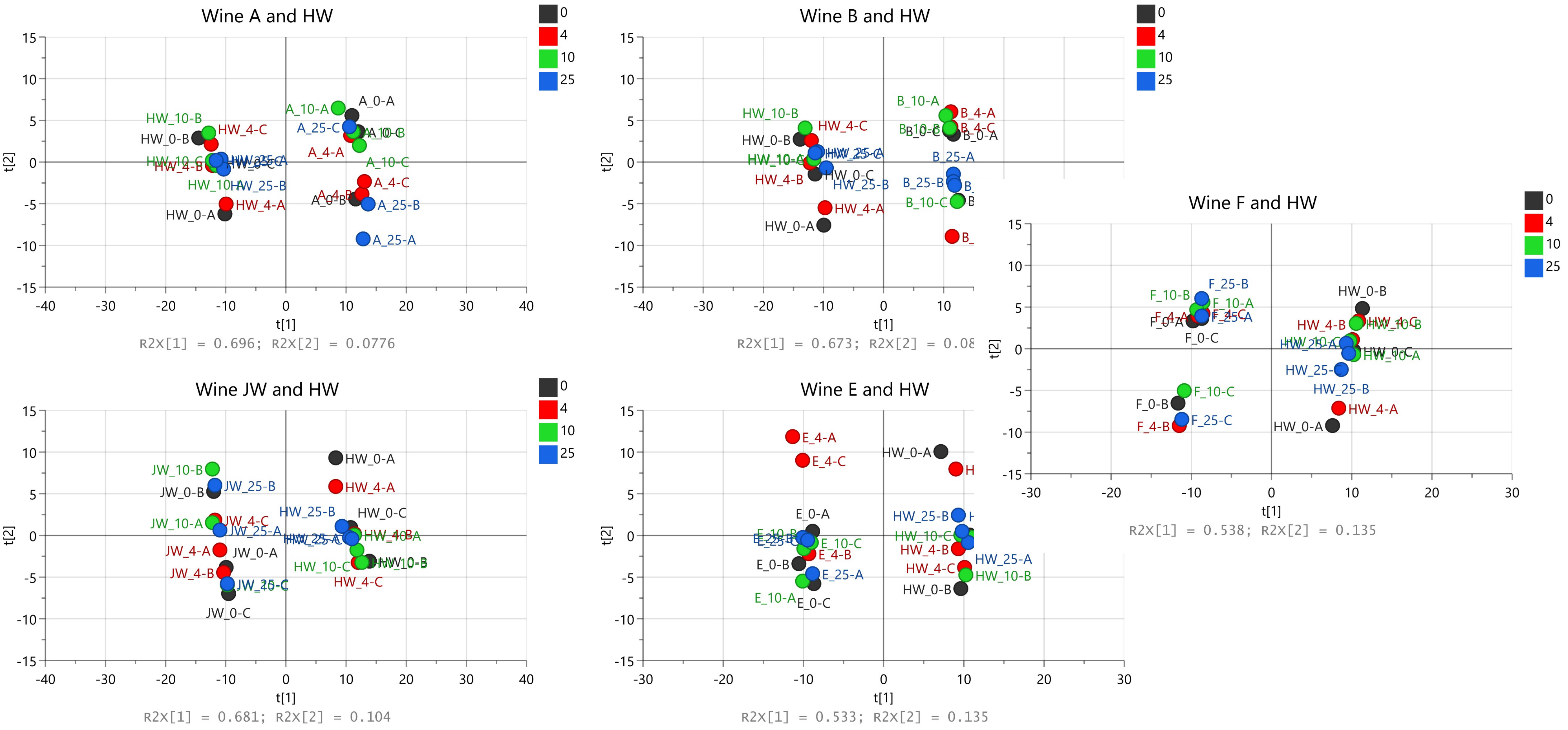

Supplemental data 2. Individual PCA analysis of 6 wines analyzed treated with chitosan and analyzed by UPLC-ESI(-) HRMS. A dataset of 224 ions, four doses of treatment (0, 4, 10 and 25 g/hL) and three replicates for each dose were considered.

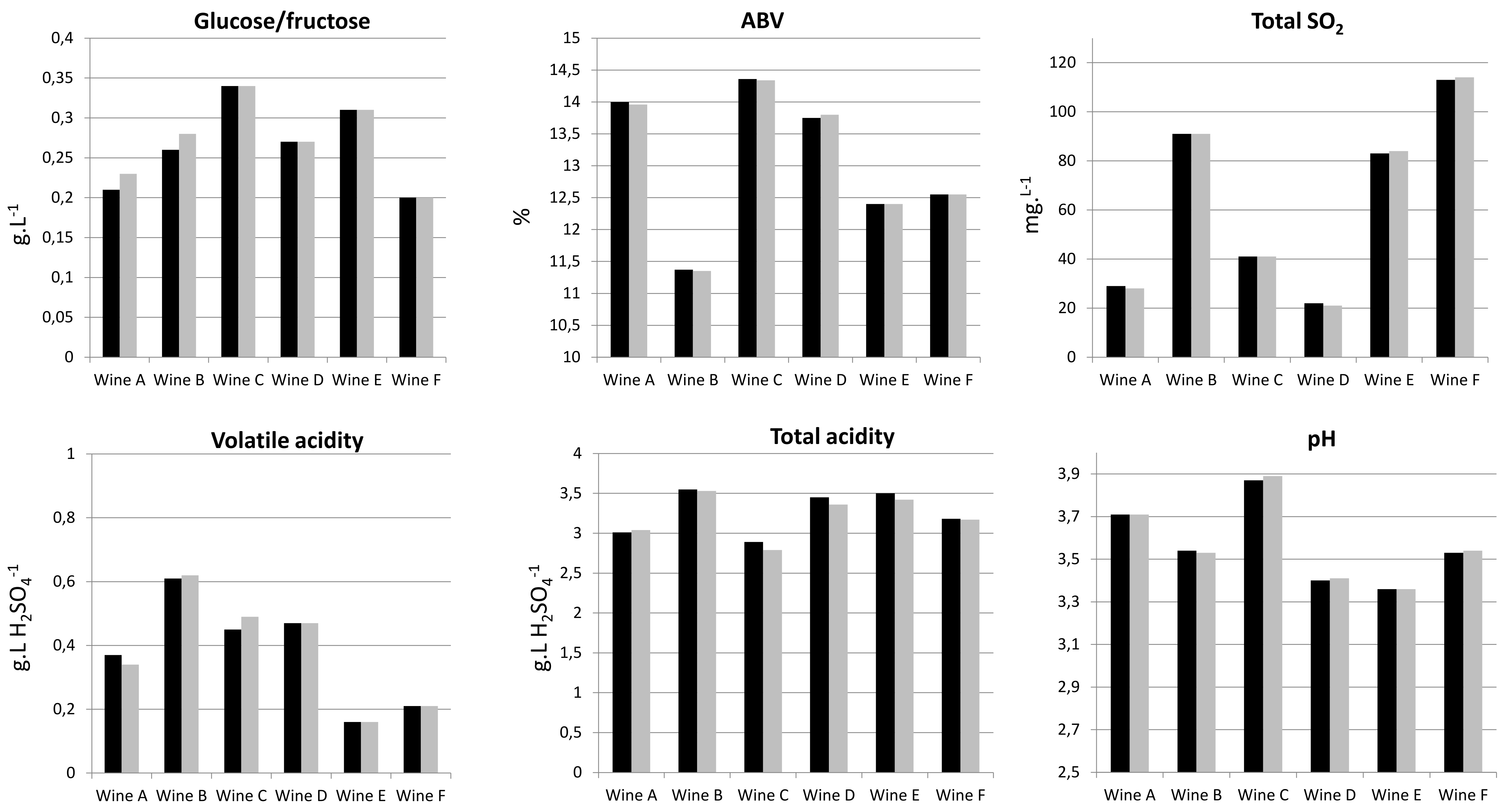

Supplemental data 3: Oenological parameters of untreated (black) and chitosan treated (10 g/L, gray) wines A to F.

For all assays, the standard deviation was below 5%.
